# Fall Risk Classification in Community-Dwelling Older Adults Using a Smart Wrist-Worn Device and the Resident Assessment Instrument-Home Care: Prospective Observational Study

**DOI:** 10.2196/12153

**Published:** 2019-06-07

**Authors:** Yang Yang, John P Hirdes, Joel A Dubin, Joon Lee

**Affiliations:** 1 Faculty of Applied Health Sciences School of Public Health and Health Systems University of Waterloo Waterloo, ON Canada; 2 Department of Statistics and Actuarial Science Faculty of Mathematics University of Waterloo Waterloo, ON Canada; 3 Department of Community Health Sciences Cumming School of Medicine University of Calgary Calgary, AB Canada; 4 Department of Cardiac Sciences Cumming School of Medicine University of Calgary Calgary, AB Canada

**Keywords:** falls, elderly, wearable devices, machine learning, interRAI

## Abstract

**Background:**

Little is known about whether off-the-shelf wearable sensor data can contribute to fall risk classification or complement clinical assessment tools such as the Resident Assessment Instrument-Home Care (RAI-HC).

**Objective:**

This study aimed to (1) investigate the similarities and differences in physical activity (PA), heart rate, and night sleep in a sample of community-dwelling older adults with varying fall histories using a smart wrist-worn device and (2) create and evaluate fall risk classification models based on (i) wearable data, (ii) the RAI-HC, and (iii) the combination of wearable and RAI-HC data.

**Methods:**

A prospective, observational study was conducted among 3 faller groups (G_0_, G_1_, G_2+_) based on the number of previous falls (0, 1, ≥2 falls) in a sample of older community-dwelling adults. Each participant was requested to wear a smart wristband for 7 consecutive days while carrying out day-to-day activities in their normal lives. The wearable and RAI-HC assessment data were analyzed and utilized to create fall risk classification models, with 3 supervised machine learning algorithms: logistic regression, decision tree, and random forest (RF).

**Results:**

Of 40 participants aged 65 to 93 years, 16 (40%) had no previous falls, whereas 8 (20%) and 16 (40%) had experienced 1 and multiple (≥2) falls, respectively. Level of PA as measured by average daily steps was significantly different between groups (*P*=.04). In the 3 faller group classification, RF achieved the best accuracy of 83.8% using both wearable and RAI-HC data, which is 13.5% higher than that of using the RAI-HC data only and 18.9% higher than that of using wearable data exclusively. In discriminating between {G_0_+G_1_} and G_2+_, RF achieved the best area under the receiver operating characteristic curve of 0.894 (overall accuracy of 89.2%) based on wearable and RAI-HC data. Discrimination between G_0_ and {G_1_+G_2+_} did not result in better classification performance than that between {G_0_+G_1_} and G_2+_.

**Conclusions:**

Both wearable data and the RAI-HC assessment can contribute to fall risk classification. All the classification models revealed that RAI-HC outperforms wearable data, and the best performance was achieved with the combination of 2 datasets. Future studies in fall risk assessment should consider using wearable technologies to supplement resident assessment instruments.

## Introduction

### Background

By definition, a fall refers to “an event which results in a person coming to rest inadvertently on the ground or floor or other lower level” [1]. The high prevalence and negative impact of falls in older people have become a serious public health issue that affects the independence of older adults, distress in caregivers, and health service utilization [2]. Due to the multifactorial nature of risk factors for falls, current fall prevention strategies are comprehensive and multifaceted [3,4]. An important goal for geriatrics and public health agencies is to accurately identify fall risks and mitigate physical and psychological harm caused by falls. In fact, falls have been used as indicators of the quality of care in home care settings [5,6].

Heart rate (HR) and heart rate variability (HRV) are hypothesized biomarkers of frailty, which implies a growing susceptibility to stressors and functional decline [7,8]. These two parameters mirror the adaptability of the heart to stressors. The study by Ogliari et al (2015) [7] examined whether HR and HRV are correlated with functional status in the aging population. Participants with the highest resting HR had increased risk of decline in performing basic activities on the Activities of Daily Living (ADL) scale and Instrumental Activities of Daily Living (IADL) tasks, with a nearly 80% and a 35% increased risk, respectively [7]. Participants with the lowest HRV had approximately a 25% increased risk of decline in performing the ADL and IADL tasks [7]. The results have shown that a higher resting HR and lower HRV in the target population was associated with poorer functional performance in daily life, as well as higher risk of functional decline [7].

Frail older people expose to great risk for serious health problems, including falls, disability, hospitalization, and mortality [9]. A functional decline and a higher level of frailty caused by the muscular atrophy would escalate the risk for falls in older population [8,10,11]. The occurrence of falls increases with frailty level [4,11]. Frailty and HRV are not only indicators of the decline in health condition [7,8,10] but also served as independent predictors for incident falls in several studies [10,11].

Various studies have shown that loss of sleep implicates a decline in the sense of balance, associating with a number of cognitive impairments such as poor concentration, memory loss, low reaction, and impaired problem solving and cognition [12-14]. It has suggested that insufficient sleep may result in risk for falls [12-16]. Short sleep duration, which accounts for habitual night sleep difficulties, is significantly associated with falls [12-16].

Evidence-based fall risk assessment can lead to proper interventions for people who are at risk for falls. To categorize subjects into faller (high risk) and nonfaller (low risk) groups, the 3 main criteria identified in the literature [17] for such classification are as follows: (1) previous history of falls, (2) prediction of future falls, and (3) clinical assessments. Several studies have incorporated a variety of independent predictors into prediction models based on clinical tests. For example, the Berg Balance Test [18], clinical- and impairment-based tests [19], neuromuscular or cognitive tests [20], the blood pressure change on upright tilting [21], depressive symptoms [22], sleep problems or urinary incontinence [16], and frailty [10,11] have been utilized to predict falls in the aging population. These clinical assessments often use assessment scores to categorize older adults into a binary outcome, that is, fallers or nonfallers [23]. However, this type of assessment oversimplifies the risk of falling in older people, which is more accurately classified by continuous fuzzy boundaries between multiple risk categories rather than a hard boundary between only two groups [23].

Recent technological advances have incorporated wearable sensor-based systems into the protocols of fall risk assessment [17,23]. A wearable sensor system can continuously monitor body movement during day-to-day activities, carried out naturally in real-life environments [17,23]. In a review of fall risk assessment in older adults with sensor-based systems, Howcroft et al (2013) [17] evaluated inertial sensors, sensor location, assessed activity, variables, and prediction models of fall risk assessment [17]. The study revealed that variables measured by sensors have the potential to predict individuals who are at risk of falling and forecast the time-to-incident [17]. Marschollek et al (2011) [23] conducted a study to compare the predictive performance between the conventional fall risk assessment and sensor-based assessment in older adults [23]. The results demonstrated that accelerometer-based fall risk model has almost the same performance as a conventional assessment model [23]. Due to the multifactorial risk factors for falls, sensor-based prediction models may provide important information to conventional assessments and are possible to perform within real-life environments at low cost [17,23].

The interRAI suite of assessment instruments [24,25] are designed to provide standardized clinical data to support care planning in a variety of clinical domains. For example, fall assessments are used to guide care and service planning in a wide range of settings, from independent residences through nursing homes and palliative care [24,26]. The Resident Assessment Instrument for Home Care (RAI-HC) is a baseline geriatric assessment to evaluate older adults who utilize home care services by assessing their needs and ability levels [5,27]. With a variety of assessment information, the RAI-HC system is composed of two key components: the Minimum Data Set-Home Care, which is the basal portion of the RAI-HC, and the Clinical Assessment Protocols [27]. In addition, various clinical scales and indices within each interRAI instrument can also be used to evaluate each client’s current health conditions (Scales: status and outcome measures). For instance, the measurement of ADL, cognition, communication, pain, behavior, and mood utilizes standardized scoring schema to generate summary indicators [26].

The interRAI assessment system is not only a suite of comprehensive and standardized assessment tools that are used in different care settings but has been utilized in several fall-related studies [18,27-44]. For example, Muir et al (2008) [18] conducted 1 prospective cohort study using the Berg Balance Scale to examine the predictive effectiveness for any fall (≥1 fall), recurrent falls (≥2 falls), and injury-related falls based on the interRAI Community Health Assessment (CHA) [18]. The CHA and RAI-HC assessments have been widely used in studies investigating the risk factors for falls [18,27,32,33], fear of falling [28-31], and the comparative analyses of nonfallers versus fallers, nonfallers and one-time fallers versus recurrent fallers [18,27,32,33].

### Objectives

To our knowledge, no prior research has combined off-the-shelf wearable sensor data with the RAI-HC assessment to examine the characteristics of different faller groups in older adults living in community, and, furthermore, to build classification models for fall risk assessment using these two data sources. This study aimed to (1) investigate the similarities and differences in physical activity (PA), HR, and night sleep patterns, which are risk factors associated with falls [7,12-14,45,46], among 3 independent older adult faller groups in community-based settings, with continuous measurements from a smart wrist-worn device and (2) create and evaluate fall risk classification models based on (i) wearable data (Wearable), (ii) the RAI-HC, and (iii) the combination of wearable and RAI-HC data (Wearable + RAI-HC). The number of previous falls was targeted as a proxy for fall risk throughout this study [18,27,32,33,47,48].

## Methods

### Study Design

Using a smart wearable device, a prospective, observational study was conducted to investigate the similarities and differences among 3 independent faller groups, that is, nonfaller (G_0_, people who have zero (0) falls in the last 90 days), single faller (G_1_, people who have 1 fall in the last 90 days), and recurrent faller (G_2+_, people who have ≥2 falls in the last 90 days) in community-based settings, in a sample of older adults living in community settings, with continuous measurements of PA, HR, and night sleep. The nonfaller, single faller, or recurrent faller stratus is within 90 days to be consistent with the standard interval of the reassessment of RAI-HC [49].

Each participant was requested to wear the Xiaomi Mi Band Pulse 1S (hereinafter referred to as the *Mi Band*) on their wrist for 7 consecutive days while carrying out day-to-day activities in their normal lives. The Mi Band is a wearable activity tracker, monitoring the activity of movements, tracing quality of sleep, and HR. It is a low-cost band, weighted 5.5 g, and comes with power-efficient accelerometer and photoelectric HR sensor [50]. Xiaomi Corporation, a Chinese electronics company headquartered in Beijing, China is the manufacturer.

The battery capacity of the Mi Band is 45 mAh [50], with approximately 30 days standby time. We tested the battery life before data collection under normal wearing condition (ie, wearing the Mi Band while carrying out day-to-day activities naturally in real-life environments), which lasted more than 15 days. Before collecting data from each participant, the battery was fully charged. To ensure that no running-out-of-battery incident occurred during data collection, participants were given instructions and demonstration on when and how to recharge the battery by themselves before data collection commence. A printout copy of the instruction was given to each participant as part of the information kit during data collection period.

A Moto E mobile phone was paired with each Mi Band wirelessly via Bluetooth to collect data, synchronize, and provide health metrics to each individual. A total of two companion apps, Mi Fit and Mi Band Tools, were installed on each mobile phone to facilitate data collection. The wearable and RAI-HC data were further analyzed to create fall risk classification models and evaluate their classification performance.

### Participant Recruitment

A sample of community-dwelling older people, who were active clients of the Waterloo Wellington Community Care Access Centre (WW CCAC) and were assessed with the RAI-HC instrument within a 1-year time window, was recruited in the Kitchener, Waterloo, Cambridge, and Guelph areas in Ontario, Canada between August and December 2016.

The inclusion criteria were that the participants must have been aged ≥65 years, living independently with or without family members at-home or community-based settings (eg, retirement home), and were able to walk without any assistive device. Individuals who have been diagnosed with end-stage disease or have been on medications of benzodiazepines, antidepressants, cardiac medications, narcotics, and anticonvulsants were excluded from participating in this study.

Informed written consent was obtained from all participants. This study was granted research ethics clearance by a University of Waterloo Research Ethics Committee. The study was also approved by the institutional review board at WW CCAC.

### Measurements

#### Number of Previous Falls

To assess the fall frequency, participants responded to the following questions upon enrollment and at the end of the wearable data collection phase: (1) “Have you fallen in the last 90 days?” (2) “How many times have you fallen in the last 90 days?” As the reassessment of RAI-HC at a standard interval is 90 days [49], we complied with this time window for the measurement of falls. Participants were categorized into G_0_, G_1_, or G_2+_ based on their self-reported number of falls at the end of the wearable data collection phase.

There was a time gap between the RAI-HC assessment and wearable data collection (mean_gap_ 107.6 days, SD 18.1 days; range –67.5-431 days). Some participants had new falls since their last RAI-HC assessments, which resulted in discrepancies between the self-reported fall frequency at wearable data collection and the corresponding assessment on the RAI-HC system. To be consistent, self-reported falls frequency at the end of the wearable data collection phase was used when analyzing wearable data only. The fall frequency on the RAI-HC assessment was used for model-building based on the RAI-HC data only as well as Wearable + RAI-HC data. In case some participants self-reported fewer number of falls than what had been reported by their primary caregivers, the higher number of falls was used in this study.

**Table 1:** Variable description of wearable data.

Variable	Description	Unit
Daily distance	Daily average distance on walking, jogging, or running	Meter
Daily steps	Daily average steps on walking, jogging, or running	Number
Daily activity time	Daily average time people spend on walking, jogging, or running	Second
Daily resting heart rate (HR)	Daily average heart beats per minute (BPM) while at complete rest (sleeping)	BPM
Daily walking HR	Daily average heart beats per minute while at walk	BPM
Daily sleep duration	Daily average duration while at sleep at night	Minute
Daily deep sleep time	Daily average duration while at deep sleep at night	Minute
Daily light sleep time	Daily average duration while at light sleep at night	Minute
Daily awake time	Daily average duration while awake at night	Minute

#### Wearable Data

Wearable sensor data collected from the Mi Band included continuous monitoring of PA, HR, and night sleep. PA and night sleep data were collected every minute, whereas HR was monitored every 2 min. By default, the Mi Band and Mi Fit app present no built-in function to extract data. A third-party script allowed data extraction via Android backup [51]. Initial wearable data were aggregated as daily averages for the analyses in this study. A list of individual variables derived from the Mi Band is presented in Table 1.

#### Resident Assessment Instrument-Home Care Data

All participants with informed written consent contributed 1 assessment each, with the latest one being selected. In this study, we used 210 variables in the RAI-HC data for analyses, including demographic information, assessment information across all the screening domains (see Multimedia Appendix 1).

### Statistical Data Analysis

Data analyses were performed using R (version 3.4.0), a free statistical software for data analysis by the R Foundation for Statistical Computing. Of the 38 variables, 40 cases and 1520 values in the wearable data, 55.3%, 65%, and 6.4% have at least 1 missing value, respectively, in terms of 1- (PA and sleep) and 2-min (HR) resolution. Of the 210 variables and 40 cases in the RAI-HC dataset, 19.8% and 100% had at least 1 missing value, respectively. Of the total of 8400 values corresponding to all combinations of the 210 variables and 40 cases, 16.3% were missing. The missing values in the RAI-HC dataset were replaced by referring to previous assessments. The missing values in the wearable data were imputed using the maximum likelihood estimates with the expectation-maximization algorithm (eg, [52]).

Descriptive statistics and simple statistical analyses were conducted to examine the similarities and differences in wearable data collected from the Mi Band from all participants. All wearable parameters (continuous variables) extracted from the Mi Band were tested for normality by using the Shapiro-Wilk test. The one-way analysis of variance (ANOVA) and Kruskal-Wallis H test were conducted to compare the means and medians of the 3 independent groups (G_0_, G_1_, and G_2+_) for normally distributed and skewed data, respectively. A two-way repeated measures ANOVA test was performed to examine the differences between groups with repeated measurements of PA, HR, and night sleep, and hence, evaluate if there was an interaction between the 7 days of measurement and groups. In all statistical analyses, *P* values ≤.05 were considered significant. However, in case of any main effect statistical significance among all groups, pairwise comparisons between groups were investigated with Bonferroni correction.

### Fall Risk Classification

To build the classification models and evaluate the classification performance of several models in classifying fall risks, a 2-step approach was employed. First, the ordinal attribute of falls (0, 1, and ≥2) within the last 90 days was used as the outcome variable, representing 3 faller groups (G_0_, G_1_, and G_2+,_ respectively), for building proportional odds models (POM). Second, the 3-class fall risk was dichotomized in two different ways: (1) grouping {G_1_+G_2+_} and comparing with G_0_ and (2) grouping {G_0_+G_1_} and comparing with G_2+_. A total of 3 supervised machine learning algorithms were utilized: logistic regression, decision tree (DT), and random forest (RF).

Given the large number of features in both datasets, there was a good chance that many of them are collinear or redundant. The multicollinearity test was conducted, and the collinear variables with a high variance inflation factor (≥5) were omitted for further analyses [53]. To identify discriminative independent variables contributing to fall frequency and to create accurate classification models, the recursive feature elimination algorithm available in the Caret R package was employed to rank-order each predictor’s importance to classification. As both the wearable and RAI-HC datasets had many variables and relatively few cases, the objective of this feature selection process was to get a total number of best subset features of no more than 10% of the sample size for the final classification models.

Classification models were trained based on (1) Wearable, (2) RAI-HC, and (3) Wearable + RAI-HC. The growing method for DT models was Classification and Regression Trees algorithm, with pruning to avoid overfitting. Key parameters included pruned, minimum child size=3, minimum parent size=5, and Gini was applied as the impurity measure. Key parameters for RF models included the number of trees grown=100, minimum size of terminal nodes=5, and the number of variables sampled at each split randomly=3. Due to the small size of training data in this study, each final model was evaluated using leave-one-out cross-validation. For the 3-class outcome, the classification accuracy, recall, precision, and F_1_ score were calculated for each final model, and the area under the receiver operating characteristic curve (AUC), accuracy, recall, precision, and F_1_ score were calculated for the dichotomized fall risks. To minimize the impact of different fall assessment at two study elements on classification performance, individuals who had an additional fall each in between the RAI-HC and wearable sensor data collection within the last 90 days’ time window were excluded for model building.

## Results

### Subject Characteristics

Of the 40 participants aged 65 to 93 years in this study, 22 (55%) were males, and 18 (45%) were females. Table 2 shows the basic characteristics of all participants in this study based on their latest RAI-HC assessments.

### Statistical Analysis

The results of the Shapiro-Wilk tests of normality showed that only the daily activity time (G_0_: *P*=.67, G_1_: *P*=.30, G_2+_: *P*=.09) was normally distributed in all 3 groups. Table 3 summarizes the PA, HR, and night sleep measurements collected by the Mi Band from different faller groups.

**Table 2:** Baseline characteristics of the participants.

Characteristics		G_0_^a^	G_1_^b^	G_2+_^c^	Total
Partcipants, n (%)		16 (40.0)	8 (20.0)	16 (40.0)	40 (100)
**Age (years), mean (SD)**		75.2 (7.5)	74.0 (6.3)	77.8 (7.4)	76.0 (7.2)
	Males	73.8 (9.8)	71.9 (2.1)	76.1 (6.5)	74.8 (7.2)
	Females	76.2 (5.6)	75.3 (7.9)	82.9 (8.6)	77.3 (7.2)
**Age group (years), n (%)**					
	65-74	8 (20.0)	7 (17.5)	6 (15.0)	21 (52.5)
	75-84	6 (15.0)	0	7 (17.5)	13 (32.5)
	85-94	2 (5.0)	1 (2.5)	3 (7.5)	6 (15.0)
**Gender, n (%)**					
	Males	7 (17.5)	3 (7.5)	12 (30.0)	22 (55.0)
	Females	9 (22.5)	5 (12.5)	4 (10.0)	18 (45.0)

^a^G_0_ people who have zero (0) falls in the last 90 days.

^b^G_1_ people who have one (1) fall in the last 90 days.

^c^G_2+_ people who have two or more (≥2) falls in the last 90 days.

**Table 3:** The measurements of wearable components by group.

Measurements	G_0_^a^	G_1_^b^	G_2+_^c^
Daily distance (meters), median (IQR^d^)	2040.7 (571.1-2643.2)	908.7 (163.4-1575.1)	490.8 (103.3-1551.2)
Daily steps, median (IQR)	3094.1 (889.4-4029.5)	1415.3 (238.1-2441.5)	768.1 (145.7-2408.6)
Daily activity time (seconds), mean (SD)	3160.2 (1725.2)	1921.4 (1264.1)	1732.4 (1670.7)
Daily resting heart rate, median (IQR)	69.6 (68.3-81.3)	78.7 (74.6-84.7)	77.7 (72.8-81.7)
Daily walking heart rate, median (IQR)	96.4 (93.4-101.1)	94.6 (91.6-105.3)	103.5 (92.2-130.0)
Daily sleep duration (min), median (IQR)	282.7 (247.8-368.3)	287.9 (144.8-428.0)	134.3 (112.8-234.8)
Daily deep sleep time (min), median (IQR)	67.7 (27.3-102.0)	69.1 (11.9-146.6)	27.1 (11.4-53.2)
Daily light sleep time (min), median (IQR)	231.4 (146.2-273.3)	200.0 (105.3-290.5)	116.0 (90.4-184.7)
Daily awake time (min), median (IQR)	21.0 (11.6-40.8)	11.9 (2.9-39.1)	6.1 (1.0-38.1)

^a^G_0_ people who have zero (0) falls in the last 90 days.

^b^G_1_ people who have one (1) fall in the last 90 days.

^c^G_2+_ people who have two or more (≥2) falls in the last 90 days.

^d^IQR: interquartile range.

#### Physical Activity Measurements

The one-way ANOVA test results showed that there was a significant difference in daily activity time (*P*=.04). However, the follow-up comparisons with the Games-Howell test indicated that the actual pairwise differences were not significant.

The Kruskal-Wallis H test results revealed that there was a significant difference in daily steps among the 3 faller groups (*P*=.04), with a mean rank daily steps of 26.53 for G_0_, 18.00 for G_1_, and 15.67 for G_2+_. The posthoc Mann-Whitney test results showed that the daily steps were not significantly different between any two comparison groups, with a Bonferroni correction at a 0.05/3=0.0167 level of significance.

Similarly, a significant difference was found in daily distance among 3 faller groups (*P*=.04), with a mean rank daily distance of 26.53, 17.92, and 15.75 for G_0_, G_1,_ and G_2+_, respectively. The posthoc Mann-Whitney tests with Bonferroni correction indicated that daily distance was not significantly different between any two comparison groups.

The two-way repeated measures ANOVA test results revealed that there was a significant main effect of steps by days between groups (*P*=.02). The posthoc tests with Bonferroni correction showed no significant pairwise differences among the 3 groups. The main effect of day of measurement was insignificant, indicating that there was no consistent difference in step counts across different days, if the groups being measured were ignored. No significant interaction effect between daily steps and the 3 faller groups was detected.

#### Heart Rate Measurements

The Kruskal-Wallis H test results indicated no significant difference in daily resting HR or daily walking HR between groups.

Furthermore, the mean, median, SD, and interquartile range (IQR) of each participant’s daily average HR was examined for differences across the groups. The results of the normality test revealed that the SD of daily average HR was normally distributed across all 3 groups. The mean, median, and IQR of daily average HR were shown to be significantly non-normal (*P*<.001, *P*<.001, and *P*=.007, respectively).

The one-way ANOVA test results showed that there was no significant difference in the participants’ SD of daily average HR. The Kruskal-Wallis H test results revealed no significant difference in the mean, median, or IQR of daily average HR between groups. The two-way repeated measures ANOVA test results revealed an insignificant main effect of HR by days between groups. The main effect of the days being measured was nonsignificant, indicating that there was no consistent difference in HR across different days, if the groups being measured were ignored. No significant interaction effect between daily average HR and the 3 faller groups was detected.

#### Night Sleep Measurements

The Kruskal-Wallis H test results revealed that there was no statistically significant difference in daily sleep duration, daily deep sleep time, daily light sleep time, or daily awake time among 3 faller groups.

The two-way repeated measures ANOVA test results showed an insignificant main effect of sleep duration by days between groups. The main effect of the days being measured was insignificant, indicating that there was no consistent difference in sleep duration across different days, if the groups being measured were ignored. No significant interaction effect between daily sleep duration and the 3 faller groups was detected.

### Three-Class Classification Models

Table 4 shows the 3-class classification results for POM, DT, or RF on Wearable, RAI-HC, and Wearable+RAI-HC. In the 3 faller group classification, RF achieved the best accuracy of 0.838 (+/-0.199), recall of 0.775 (+/-0.233), precision of 0.730 (+/-0.259), and F_1_ score of 0.748 (+/-0.248) using both wearable and RAI-HC data. The lowest accuracy occurred in POM using wearable data.

**Table 4:** Three-class classification results for proportional odds models, decision tree, and random forest on Wearable, Resident Assessment Instrument-Home Care, and Wearable+Resident Assessment Instrument-Home Care.

Dataset and classifier		Accuracy	Recall	Precision	F_1_
**Wearable**					
	POM^a^	0.378 (+/-0.198)	0.351 (+/-0.177)	0.267 (+/-0.147)	0.286 (+/-0.151)
	DT^b^	0.595 (+/-0.184)	0.559 (+/-0.156)	0.389 (+/-0.186)	0.443 (+/-0.184)
	RF^c^	0.649 (+/-0.166)	0.622 (+/-0.215)	0.550 (+/-0.242)	0.568 (+/-0.223)
**RAI-HC^d^**					
	POM	0.568 (+/-0.211)	0.509 (+/-0.216)	0.417 (+/-0.238)	0.449 (+/-0.229)
	DT	0.703 (+/-0.218)	0.649 (+/-0.232)	0.554 (+/-0.299)	0.581 (+/-0.270)
	RF	0.703 (+/-0.288)	0.662 (+/-0.299)	0.649 (+/-0.321)	0.634 (+/-0.314)
**Wearable + RAI-HC**					
	POM	0.676 (+/-0.170)	0.626 (+/-0.195)	0.593 (+/-0.195)	0.584 (+/-0.191)
	DT	0.757 (+/-0.221)	0.703 (+/-0.254)	0.643 (+/-0.275)	0.662 (+/-0.266)
	RF	0.838 (+/-0.199)	0.775 (+/-0.233)	0.730 (+/-0.259)	0.748 (+/-0.248)

^a^POM: proportional odds model.

^b^DT: decision tree.

^c^RF: random forest.

^d^RAI-HC: Resident Assessment Instrument-Home Care.

### Binary Classification Models for G_0_ Versus {G_1_+G_2_+} and {G_0_+G_1_} Versus G_2+_

Table 5 tabulates the feature analysis results for all classification models, listing various features that have been selected in the 3 datasets with 3-class classification and dichotomization in two different ways. Table 6 and Table 7 list the binary classification results for the POM, DT, or RF on Wearable, RAI-HC, and Wearable+RAI-HC, utilizing two different ways of dichotomizing the 3-class outcome. In terms of binary classification models for {G_0_+G_1_} versus G_2+_, RF achieved the best AUC of 0.894 (+/-0.155), overall accuracy of 0.892 (+/-0.160), recall of 0.908 (+/-0.135), precision of 0.928 (+/-0.106), and F_1_ score of 0.888 (+/-0.166) based on Wearable + RAI-HC. The AUCs of RF based on RAI-HC and Wearable data exclusively were 0.836 (+/-0.206) and 0.795 (+/-0.247), overall accuracies of 0.838 (+/-0.192) and 0.784 (+/-0.276), respectively; whereas for G_0_ versus {G_1_+G_2+_}, RF achieved the best AUC of 0.865 (+/-0.125), overall accuracy of 0.865 (+/-0.132) based on Wearable + RAI-HC dataset. The AUCs of RF on RAI-HC and Wearable exclusively were 0.858 (+/-0.160) and 0.757 (+/-0.250), overall accuracies of 0.865 (+/-0.145) and 0.784 (+/-0.236), respectively.

**Table 5:** Feature analyses for all classification models.

Dataset	Three-class		G_0_ versus {G_1_+G_2+_}		{G_0_+G_1_} versus G_2+_	
	Top 4 features	No. of times being selected in each LOOCV^a^ iteration, n (%)	Top 4 features	No. of times being selected in each LOOCV iteration, n (%)	Top 4 features	No. of times being selected in each LOOCV iteration, n (%)
Wearable	Daily walking HR^b^	37 (100)	Daily steps	37 (100)	Daily walking HR	37 (100)
	Daily steps	36 (97.3)	Daily walking HR	37 (100)	Daily sleep duration	26 (70.3)
	Daily sleep duration	27 (73)	Median of daily avg. HR	36 (97.3)	Daily resting HR	23 (62.2)
	SD of daily avg. HR	14 (37.8)	Daily resting HR	17 (45.9)	Daily light sleep	23 (62.2)
RAI-HC^c^	MAPLe^d^	36 (97.3)	MAPLe	37 (100)	MAPLe	37 (100)
	No. of ER^e^ Visits	31 (83.8)	IADL^f^-difficulty prep meal	34 (91.9)	No. of ER Visits	35 (94.6)
	IADL-difficulty prep meal	29 (80)	Psychiatric diagnosis	33 (89.2)	Short-term memory	27 (73.0)
	Psychiatric diagnosis	20 (57.5)	Overall change in care needs	27 (73.0)	Oral-problem chewing	10 (27.0)
Wearable + RAI-HC	MAPLe	34 (91.9)	MAPLe	36 (97.3)	MAPLe	35 (94.6)
	No. of ER visits	33 (89.2)	IADL-difficulty prep meal	32 (86.5)	No. of ER visits	32 (86.5)
	IADL-difficulty prep meal	33 (89.2)	Overall change in care needs	25 (67.6)	Daily walking HR	32 (86.5)
	Daily steps	33 (89.2)	Daily steps	24 (64.9)	Short-term memory	22 (59.5)

^a^LOOCV: leave-one-out cross-validation.

^b^HR: heart rate.

^c^RAI-HC: Resident Assessment Instrument-Home Care.

^d^MAPLe: The Method for Assigning Priority Levels.

^e^ER: emergency room.

^f^IADL: instrumental activities of daily living.

**Table 6:** Classification results for binary classification models G_0_. versus {G_1_+G_2+_}.

Dataset and classifier		AUC^a^	Accuracy	Recall	Precision	F_1_
**Wearable**						
	LR^b^	0.680 (+/-0.323)	0.622 (+/-0.215)	0.604 (+/-0.222)	0.553 (+/-0.289)	0.552 (+/-0.250)
	DT^c^	0.725 (+/-0.183)	0.757 (+/-0.139)	0.725 (+/-0.183)	0.676 (+/-0.278)	0.670 (+/-0.230)
	RF^d^	0.757 (+/-0.250)	0.784 (+/-0.236)	0.757 (+/-0.250)	0.729 (+/-0.325)	0.720 (+/-0.294)
**RAI-HC^e^**							
	LR	0.840 (+/-0.236)	0.730 (+/-0.213)	0.689 (+/-0.249)	0.644 (+/-0.304)	0.648 (+/-0.276)
	DT	0.856 (+/-0.153)	0.865 (+/-0.132)	0.856 (+/-0.153)	0.868 (+/-0.181)	0.836 (+/-0.179)
	RF	0.858 (+/-0.160)	0.865 (+/-0.145)	0.858 (+/-0.160)	0.870 (+/-0.186)	0.836 (+/-0.189)
**Wearable + RAI-HC**						
	LR	0.743 (+/-0.251)	0.784 (+/-0.224)	0.766 (+/-0.232)	0.778 (+/-0.255)	0.755 (+/-0.246)
	DT	0.842 (+/-0.229)	0.865 (+/-0.192)	0.851 (+/-0.213)	0.886 (+/-0.202)	0.849 (+/-0.214)
	RF	0.865 (+/-0.125)	0.865 (+/-0.132)	0.865 (+/-0.125)	0.908 (+/-0.094)	0.853 (+/-0.139)

^a^AUC: area under the receiver operating characteristic curve.

^b^LR: logistic regression.

^c^DT: decision tree.

^d^RF: random forest.

^e^RAI-HC: Resident Assessment Instrument-Home Care.

**Table 7:** Classification results for binary classification models {G_0_+G_1_} versus G_2+_

Dataset and classifier		AUC^a^	Accuracy	Recall	Precision	F_1_
**Wearable**						
	LR^b^	0.599 (+/-0.306)	0.730 (+/-0.185)	0.599 (+/-0.252)	0.518 (+/-0.294)	0.551 (+/-0.275)
	DT^c^	0.768 (+/-0.209)	0.730 (+/-0.184)	0.750 (+/-0.210)	0.682 (+/-0.262)	0.678 (+/-0.238)
	RF^d^	0.795 (+/-0.247)	0.784 (+/-0.276)	0.795 (+/-0.247)	0.732 (+/-0.325)	0.742 (+/-0.307)
**RAI-HC^e^**						
	LR	0.842 (+/-0.306)	0.649 (+/-0.156)	0.610 (+/-0.174)	0.514 (+/-0.264)	0.524 (+/-0.214)
	DT	0.836 (+/-0.149)	0.811 (+/-0.187)	0.836 (+/-0.149)	0.836 (+/-0.217)	0.799 (+/-0.206)
	RF	0.836 (+/-0.206)	0.838 (+/-0.192)	0.858 (+/-0.175)	0.869 (+/-0.165)	0.836 (+/-0.195)
**Wearable + RAI-HC**						
	LR	0.838 (+/-0.234)	0.703 (+/-0.172)	0.676 (+/-0.200)	0.657 (+/-0.281)	0.626 (+/-0.231)
	DT	0.858 (+/-0.160)	0.838 (+/-0.200)	0.869 (+/-0.154)	0.851 (+/-0.226)	0.829 (+/-0.218)
	RF	0.894 (+/-0.155)	0.892 (+/-0.160)	0.908 (+/-0.135)	0.928 (+/-0.106)	0.888 (+/-0.166)

^a^AUC: area under the receiver operating characteristic curve.

^b^LR: logistic regression.

^c^DT: decision tree.

^d^RF: random forest.

^e^RAI-HC: Resident Assessment Instrument-Home Care.

## Discussion

### General Discussions

To the best of our knowledge, no prior study has combined off-the-shelf wearable sensor data with the interRAI assessment system to examine the characteristics of different faller groups in community-dwelling older people, or to build fall risk classification models with the combination of wearable and interRAI data. There was a gap in knowledge necessary to understand the associations between PA, HR, and night sleep and different fall frequencies in the target population. This pilot study aimed to fill this gap.

It was hypothesized that there were differences in PA, HR, and night sleep among the two faller groups in the target population. The statistical test results revealed a significant difference of PA, including daily steps, daily distance, and daily activity time between groups. The findings are consistent with the literature regarding PA and falls, that is, the decline in PA is associated with increased occurrences of falls [45,54]. However, the small sample size could have made it difficult to detect significant associations. Although there were group differences, the subsequent pairwise comparisons were not significant.

The findings in this study are in line with previous research that examined risk factors for falls in community-dwelling older adults [27,28,32,33,47,54]. For example, Gaßmann et al (2009) [47] examined predictors for single and recurrent fallers in older people living in community, and the results indicated poor health status, lower physical functioning, and mobility were risk factors for falls [47]. In our study, the top features (Table 5) incorporated into model-building were associated with poor health status, such as number of emergency room (ER) visits and IADL from the RAI-HC data, which are major risk factors for falls. As a baseline geriatric assessment to evaluate older adults who utilize home care services, the RAI-HC data represent a comprehensive assessment framework, which may serve well as a fall risk screening method. Similarly, wearable data contain discriminatory power in classifying fall risks. For instance, daily resting HR derived from the wearable device was associated with frailty, which was considered a risk factor for falls [7].

In the 3 faller group classification, RF achieved the best accuracy of using both wearable and RAI-HC data. It reveals that to achieve the best accuracy for classifying an individual into 1 of the 3 faller groups (G_0_, G_1_, or G_2+_), applying the RF algorithm on both wearable and RAI-HC data outperforms all the other methods (Table 4). Considering dichotomization of the 3-class outcome, the combination of wearable and RAI-HC data led to the best classification results as well (Table 6 and Table 7). The 2 datasets represent distinct features associated with fall risk. For example, the wearable data provide objective information on motion, whereas the RAI-HC data represent a comprehensive geriatric assessment, measuring IADL, cognition, communication, pain, behavior, and mood utilizing standardized scoring schema to generate summary indicators [26]. The merging of these 2 datasets seems to bring in added value while conducting automatic feature selection with the recursive feature elimination algorithm.

Although dichotomizing to binary classification models, the RF algorithm with both wearable, and RAI-HC data led to a strong discrimination with the AUC of 0.894, whereas classifying an individual into nonfallers and single-fallers {G_0_+G_1_} or recurrent fallers G_2+_. It is recommended to use both datasets as Table 7 suggests, and the best features are the method for assigning priority levels (MAPLe), number of ER visits, daily walking HR, and short-term memory as tabulated in Table 5. Similarly, comparing with all the methods and models that classify an individual into nonfallers G_0_ and fallers {G_1_+G_2+_}, the RF algorithm with both wearable, and RAI-HC data gave a strong discrimination with the AUC of 0.865 (Table 6). Again, it is recommended to use the combination of wearable and RAI-HC data; the best features are MAPLe, IADL-difficulty prep meal, overall change in care needs, and daily steps as tabulated in Table 5.

Comparing the two different ways of dichotomization, that is, G_0_ versus {G_1_+G_2+_} and {G_0_+G_1_} versus G_2+_, the classification models distinguishing {G_0_+G_1_} and G_2+_ had better performance. However, the binary classification results of this study did not show any consistent trend as to whether G_1_ is more similar to G_0_ or G_2+_. There seems to be no clear and hard boundary between any two adjacent groups. Intuitively, because of the multifactorial nature of risk factors for falls, the boundaries on both sides of G_1_ are expected to be fuzzy.

### Limitations

The main limitation of this study is the relatively small sample size, which is not robust to analyze the binary and accidental data of falls, especially in a machine learning context. The small number of participants compromise the accuracy and, therefore, the validity of this study findings. Although it may be difficult to generalize or draw conclusions relying on a small dataset, the leave-one-out cross validation method helps address the limitation of small dataset size. The gap between the wearable and RAI-HC data collection and the subsequent decision of using the fall frequency on the RAI-HC assessment for model-building on Wearable + RAI-HC data may have limited the true ability to compare various classifier performance between the groups. In particular, the wearable component may have been disadvantaged by correlating with outdated number of falls. Evidence suggested a response bias, in particular, social desirability bias may be introduced into this study, as some participants underreported their fall frequencies while compared with the responses from their primary caregivers. We used cross sectional data instead of longitudinal outcomes, which is another major limitation that has to be addressed in future work. The findings from this study suffer from limited generalizability because of the homogenous and small sample from community-based settings within a particular geographic area. Using retrospective fall occurrence and lack of follow-up observation accounts for another limitation. In addition, although the selected wearable device is capable of monitoring sleep patterns at night with auto sleep detection, it cannot reliably detect relatively short periods of sleep or fragmented sleep. As such, the Mi Band in this study did not properly identify daytime napping.

### Conclusions

This study provides a knowledge base that future research in fall risk assessment can leverage. By obtaining a better and fuller understanding of fall risk and varying characteristics of older people with different fall histories, more suggestions that are informed can be made for individuals in this population. Both wearable data and the RAI-HC assessment can contribute to fall risk classification. All the classification models revealed that RAI-HC outperforms wearable data and the best performance was achieved with the combination of 2 datasets. Future studies in fall risk assessment should consider using wearable technologies to supplement resident assessment instruments. Future studies are needed to work around the limitations of this study. For instance, larger sample sizes, reduced gap between the RAI-HC and wearable sensor collection, longer study periods, and possibly fuller use of the collected longitudinal data may be helpful in better estimating fall risk classification performance. Studies on different older adult populations are warranted, including clinical inpatients, long-term care, or other institutional residents.

## Appendix

Multimedia Appendix 1 List of the Resident Assessment Instrument-Home Care data variables used in this study.

## Abbreviations

ADL: Activities of Daily Living
ANOVA: analysis of variance
AUC: area under the receiver operating characteristic curve
CHA: Community Health Assessment
DT: decision tree
HR: heart rate
HRV: heart rate variability
IADL: Instrumental Activities of Daily Living
IQR: interquartile range
PA: physical activity
RAI-HC: Resident Assessment Instrument-Home Care
RF: random forest
WW CCAC: Waterloo Wellington Community Care Access Centre
