# Supplementary material for: Fall Risk Classification in Community-Dwelling Older Adults Using a Smart Wrist-Worn Device and the Resident Assessment Instrument-Home Care: Prospective Observational Study
Source: JMIR Aging. 2019 Jun 7;2(1):e12153. doi: 10.2196/12153 (PMC6716444; doi:10.2196/12153)
Supplement: Multimedia Appendix 1 [file aging_v2i1e12153_app1.docx]

**Appendix A: List of the RAI-HC data variables used in this study**

| FALLS FREQUENCY (variable of interest) |
| --- |
| **SECTION BB. PERSONAL ITEMS** |
| AGE |
| SEX |
| MARITAL STATUS |
| LANGUAGE - Interpreter needed |
| EDUCATION (Highest Level Completed) |
| RESPONSIBILITY FOR PAYMENT - Worker’s Compensation Board (WCB/WSIB) |
| **SECTION CC. REFERRAL ITEMS** |
| REASON FOR REFERRAL |
| UNDERSTANDING OF GOALS OF CARE - Skilled nursing treatments |
| UNDERSTANDING OF GOALS OF CARE - Monitoring to avoid clinical complications |
| UNDERSTANDING OF GOALS OF CARE - Rehabilitation |
| UNDERSTANDING OF GOALS OF CARE - Client/family education |
| UNDERSTANDING OF GOALS OF CARE - Family respite |
| TIME SINCE LAST HOSPITAL STAY |
| WHERE LIVED AT TIME OF REFERRAL |
| WHO LIVED WITH AT REFERRAL |
| RESIDENTIAL HISTORY |
| **SECTION A. ASSESSMENT INFORMATION** |
| REASON FOR ASSESSMENT |
| **SECTION B. COGNITIVE PATTERNS** |
| MEMORY RECALL ABILITY - Short-term memory OK |
| MEMORY RECALL ABILITY - Procedural memory OK |
| COGNITIVE SKILLS FOR DAILY DECISIONMAKING - How well client made decisions about organizing the day |
| COGNITIVE SKILLS FOR DAILY DECISIONMAKING - Worsening of decision making as compared to status of 90 DAYS AGO (or since last assessment if less than 90 days) |
| INDICATORS OF DELIRIUM - In the LAST 90 DAYS (or since last assessment if less than 90 days), client has become agitated or disoriented such that his or her safety is endangered or client requires protection by others |
| **SECTION C. COMMUNICATION/HEARING PATTERNS** |
| HEARING |
| MAKING SELF UNDERSTOOD (Expression) |
| ABILITY TO UNDERSTAND OTHERS (Comprehension) |
| COMMUNICATION DECLINE |
| **SECTION D. VISION PATTERNS** |
| VISION |
| VISUAL LIMITATION/ DIFFICULTIES |
| VISION DECLINE |
| **SECTION E. MOOD AND BEHAVIOUR PATTERNS** |
| INDICATORS OF DEPRESSION, ANXIETY, SAD MOOD - A FEELING OF SADNESS OR BEING DEPRESSED |
| INDICATORS OF DEPRESSION, ANXIETY, SAD MOOD - PERSISTENT ANGER WITH SELF OR OTHERS |
| INDICATORS OF DEPRESSION, ANXIETY, SAD MOOD - EXPRESSIONS OF WHAT APPEAR TO BE UNREALISTIC FEARS |
| INDICATORS OF DEPRESSION, ANXIETY, SAD MOOD - REPETITIVE HEALTH COMPLAINTS |
| INDICATORS OF DEPRESSION, ANXIETY, SAD MOOD - REPETITIVE ANXIOUS COMPLAINTS, CONCERNS |
| INDICATORS OF DEPRESSION, ANXIETY, SAD MOOD - SAD, PAINED, WORRIED FACIAL EXPRESSIONS |
| INDICATORS OF DEPRESSION, ANXIETY, SAD MOOD - RECURRENT CRYING, TEARFULNESS |
| INDICATORS OF DEPRESSION, ANXIETY, SAD MOOD - WITHDRAWAL FROM ACTIVITIES OF INTEREST |
| INDICATORS OF DEPRESSION, ANXIETY, SAD MOOD - REDUCED SOCIAL INTERACTION |
| MOOD DECLINE |
| BEHAVIOURAL SYMPTOMS - WANDERING |
| BEHAVIOURAL SYMPTOMS - VERBALLY ABUSIVE BEHAVIOURAL SYMPTOMS |
| BEHAVIOURAL SYMPTOMS - PHYSICALLY ABUSIVE BEHAVIOURAL SYMPTOM |
| BEHAVIOURAL SYMPTOMS - SOCIALLY INAPPROPRIATE/ DISRUPTIVE BEHAVIOURAL SYMPTOMS |
| BEHAVIOURAL SYMPTOMS - RESISTS CARE |
| CHANGES IN BEHAVIOUR SYMPTOMS |
| **SECTION F. SOCIAL FUNCTIONING** |
| INVOLVEMENT - At ease interacting with others |
| INVOLVEMENT - Openly expresses conflict or anger with family/friends |
| CHANGE IN SOCIAL ACTIVITIES |
| ISOLATION - Length of time client is alone during the day |
| ISOLATION - Client says or indicates that he/she feels lonely |
| **SECTION G. INFORMAL SUPPORT SERVICES** |
| TWO KEY INFORMAL HELPERS - Primary lives with client |
| TWO KEY INFORMAL HELPERS - Secondary lives with client |
| TWO KEY INFORMAL HELPERS - Primary relationship to client |
| TWO KEY INFORMAL HELPERS - Secondary relationship to client |
| TWO KEY INFORMAL HELPERS - Primary advice or emotional support |
| TWO KEY INFORMAL HELPERS - Secondary advice or emotional support |
| TWO KEY INFORMAL HELPERS - Primary IADL care |
| TWO KEY INFORMAL HELPERS - Secondary IADL care |
| TWO KEY INFORMAL HELPERS - Primary ADL care |
| TWO KEY INFORMAL HELPERS - Secondary ADL care |
| TWO KEY INFORMAL HELPERS - Primary emotional support |
| TWO KEY INFORMAL HELPERS - Secondary emotional support |
| TWO KEY INFORMAL HELPERS - Primary IADL care |
| TWO KEY INFORMAL HELPERS - Secondary IADL care |
| TWO KEY INFORMAL HELPERS - Primary ADL care |
| TWO KEY INFORMAL HELPERS - Secondary ADL care |
| CAREGIVER STATUS - A caregiver is unable to continue in caring activities |
| CAREGIVER STATUS - Primary caregiver is not satisfied with support received from family and friends |
| CAREGIVER STATUS - Primary caregiver expresses feelings of distress, anger or depression |
| CAREGIVER STATUS - NONE OF ABOVE |
| EXTENT OF INFORMAL HELP (HOURS OF CARE, ROUNDED) - Sum of time across five weekdays |
| EXTENT OF INFORMAL HELP (HOURS OF CARE, ROUNDED) - Sum of time across two weekend days |
| **SECTION H. PHYSICAL FUNCTIONING: ● IADL PERFORMANCE IN 7 DAYS ● ADL PERFORMANCE IN 3 DAYS** |
| IADL SELF-PERFORMANCE - MEAL PREPARATION (Performance) |
| IADL SELF-PERFORMANCE - MEAL PREPARATION (Difficulty) |
| IADL SELF-PERFORMANCE - ORDINARY HOUSEWORK (Performance) |
| IADL SELF-PERFORMANCE - ORDINARY HOUSEWORK (Difficulty) |
| IADL SELF-PERFORMANCE - MANAGING FINANCES (Performance) |
| IADL SELF-PERFORMANCE - MANAGING FINANCES (Difficulty) |
| IADL SELF-PERFORMANCE - MANAGING MEDICATIONS (Performance) |
| IADL SELF-PERFORMANCE - MANAGING MEDICATIONS (Difficulty) |
| IADL SELF-PERFORMANCE - PHONE USE (Performance) |
| IADL SELF-PERFORMANCE - PHONE USE (Difficulty) |
| IADL SELF-PERFORMANCE - SHOPPING (Performance) |
| IADL SELF-PERFORMANCE - SHOPPING (Difficulty) |
| IADL SELF-PERFORMANCE - TRANSPORTATION (Performance) |
| IADL SELF-PERFORMANCE - TRANSPORTATION (Difficulty) |
| ADL SELF-PERFORMANCE - MOBILITY IN BED |
| ADL SELF-PERFORMANCE - TRANSFER |
| ADL SELF-PERFORMANCE - LOCOMOTION IN HOME |
| ADL SELF-PERFORMANCE - LOCOMOTION OUTSIDE OF HOME |
| ADL SELF-PERFORMANCE - DRESSING UPPER BODY |
| ADL SELF-PERFORMANCE - DRESSING LOWER BODY |
| ADL SELF-PERFORMANCE - EATING |
| ADL SELF-PERFORMANCE - TOILET USE |
| ADL SELF-PERFORMANCE - PERSONAL HYGIENE |
| ADL SELF-PERFORMANCE - BATHING |
| ADL SELF-PERFORMANCE - ADL DECLINE |
| ADL SELF-PERFORMANCE - PRIMARY MODES OF LOCOMOTION (Indoors) |
| ADL SELF-PERFORMANCE - PRIMARY MODES OF LOCOMOTION (Outdoors) |
| ADL SELF-PERFORMANCE - STAIR CLIMBING |
| ADL SELF-PERFORMANCE - STAMINA (number of days client usually went out of the house or building in which client lives) |
| ADL SELF-PERFORMANCE - STAMINA (Hours of physical activities in the last 3 days) |
| ADL SELF-PERFORMANCE - FUNCTIONAL POTENTIAL (Client believes he/she capable of increased functional independence (ADL, IADL, mobility)) |
| ADL SELF-PERFORMANCE - FUNCTIONAL POTENTIAL (Caregivers believe client is capable of increased functional independence (ADL, IADL, mobility)) |
| ADL SELF-PERFORMANCE - FUNCTIONAL POTENTIAL (Good prospects of recovery from current disease or conditions, improved health status expected) |
| ADL SELF-PERFORMANCE - FUNCTIONAL POTENTIAL (NONE OF ABOVE) |
| **SECTION I. CONTINENCE IN LAST 7 DAYS** |
| BLADDER CONTINENCE - In LAST 7 DAYS (or since last assessment if less than 7 days) control of urinary bladder function |
| BLADDER CONTINENCE - Worsening of bladder incontinence as compared to status 90 days ago |
| BLADDER DEVICES - Use of pads or briefs to protect against wetness |
| BLADDER DEVICES - Use of an indwelling urinary catheter |
| BLADDER DEVICES - NONE OF ABOVE |
| BOWEL CONTINENCE |
| **SECTION J. DISEASE DIAGNOSES** |
| Cerebrovascular accident (stroke) |
| Congestive heart failure |
| Coronary artery disease |
| Hypertension |
| Alzheimer’s |
| Dementia other than Alzheimer's disease |
| Arthritis |
| Other fractures |
| Osteoporosis |
| Cataract |
| Any psychiatric diagnosis |
| Cancer (in past 5 years) not including skin cancer |
| Diabetes |
| Emphysema/ COPD/ asthma |
| Thyroid disease (hyper or hypo) |
| **SECTION K. HEALTH CONDITIONS AND PREVENTIVE HEALTH MEASURES** |
| PREVENTIVE HEALTH (PAST TWO YEARS) - Blood pressure measured |
| PREVENTIVE HEALTH (PAST TWO YEARS) - Received influenza vaccination |
| PREVENTIVE HEALTH (PAST TWO YEARS) - Test for blood in stool or screening endoscopy |
| PREVENTIVE HEALTH (PAST TWO YEARS) - IN FEMALE: Received breast examination or mammography |
| PROBLEM CONDITIONS PRESENT ON 2 OR MORE DAYS - Diarrhea |
| PROBLEM CONDITIONS PRESENT ON 2 OR MORE DAYS - Difficulty urinating or urinating 3 or more times at night |
| PROBLEM CONDITIONS PRESENT ON 2 OR MORE DAYS - Loss of appetite |
| PROBLEM CONDITIONS PRESENT ON 2 OR MORE DAYS - Vomiting |
| PROBLEM CONDITIONS PRESENT ON 2 OR MORE DAYS - NONE OF ABOVE |
| PROBLEM CONDITIONS - Chest pain/pressure at rest or on exertion |
| PROBLEM CONDITIONS - Dizziness or lightheadedness |
| PROBLEM CONDITIONS - Edema |
| PROBLEM CONDITIONS - Shortness of breath |
| PROBLEM CONDITIONS - Hallucinations |
| PROBLEM CONDITIONS - NONE OF ABOVE |
| PAIN - Frequency with which client complains or shows evidence of pain |
| PAIN - Intensity of pain |
| PAIN - From client’s point of view, pain intensity disrupts usual activities |
| PAIN - Character of pain |
| PAIN - From client’s point of view, medications adequately control pain |
| DANGER OF FALL - Unsteady gait |
| DANGER OF FALL - Client limits going outdoors due to fear of falling |
| LIFESTYLE (Drinking/ Smoking) - In the LAST 90 DAYS (or since last assessment if less than 90 days), client felt the need or was told by others to cut down on drinking, or others were concerned with client’s drinking |
| LIFESTYLE (Drinking/ Smoking) - Smoked or chewed tobacco daily |
| HEALTH STATUS INDICATORS - Client feels he/she is poor health |
| HEALTH STATUS INDICATORS - Has conditions or diseases that make cognition, ADL, mood, or behaviour patterns unstable |
| HEALTH STATUS INDICATORS - Experiencing a flare-up of a recurrent or chronic problem |
| HEALTH STATUS INDICATORS - Treatments changed in LAST 30 DAYS (or since last assessment if less than 30 days) because of a new acute episode or condition |
| HEALTH STATUS INDICATORS - NONE OF ABOVE |
| **SECTION L. NUTRITION/HYDRATION STATUS** |
| WEIGHT - Unintended weight loss of 5% or more in the LAST 30 DAYS (or 10% or more in the LAST 180 DAYS) |
| WEIGHT - Morbid obesity |
| CONSUMPTION - In at least 2 of the last 3 days, ate one or fewer meals a day |
| CONSUMPTION - Enteral tube feeding |
| SWALLOWING |
| **SECTION M. DENTAL STATUS (ORAL HEALTH)** |
| ORAL STATUS - Problem chewing |
| ORAL STATUS - Mouth is “dry” when eating a meal |
| ORAL STATUS - Problem brushing teeth or dentures |
| ORAL STATUS - NONE OF ABOVE |
| **SECTION N. SKIN CONDITION** |
| SKIN PROBLEMS - Any troubling conditions or changes in skin condition |
| ULCERS - Pressure ulcer |
| ULCERS - Stasis ulcer |
| OTHER SKIN PROBLEMS REQUIRING TREATMENT - Open lesions other than ulcers, rashes, cuts |
| OTHER SKIN PROBLEMS REQUIRING TREATMENT - Skin tears or cuts |
| OTHER SKIN PROBLEMS REQUIRING TREATMENT - Surgical wound |
| OTHER SKIN PROBLEMS REQUIRING TREATMENT - Corns, calluses, structural problems, infections, fungi |
| OTHER SKIN PROBLEMS REQUIRING TREATMENT - NONE OF ABOVE |
| HISTORY OF RESOLVED PRESSURE ULCERS - Client previously had (at any time) or has an ulcer anywhere on the body |
| WOUND/ ULCER CARE - Antibiotics, systemic or topical |
| WOUND/ ULCER CARE - Dressings |
| WOUND/ ULCER CARE - Surgical wound care |
| WOUND/ ULCER CARE - Other wound/ulcer care |
| WOUND/ ULCER CARE - NONE OF ABOVE |
| **SECTION O. ENVIRONMENTAL ASSESSMENT** |
| HOME ENVIRONMENT - Lighting in evening |
| HOME ENVIRONMENT - Flooring and carpeting |
| HOME ENVIRONMENT - Kitchen |
| HOME ENVIRONMENT - Access to home |
| HOME ENVIRONMENT - Access to rooms in house |
| HOME ENVIRONMENT - NONE OF ABOVE |
| LIVING ARRANGEMENT - As compared to 90 DAYS AGO (or since last assessment), client now lives with other persons |
| LIVING ARRANGEMENT - Client or primary caregiver feels that client would be better off in another living environment |
| **SECTION P. SERVICE UTILIZATION (IN LAST 7 DAYS)** |
| SPECIAL TREATMENTS, THERAPIES, PROGRAMS - Oxygen |
| SPECIAL TREATMENTS, THERAPIES, PROGRAMS - Medical alert bracelet or electronic security alert |
| SPECIAL TREATMENTS, THERAPIES, PROGRAMS - Special diet |
| SPECIAL TREATMENTS, THERAPIES, PROGRAMS - NONE OF ABOVE |
| MANAGEMENT OF EQUIPMENT (In Last 3 Days) - Oxygen |
| MANAGEMENT OF EQUIPMENT (In Last 3 Days) - IV |
| MANAGEMENT OF EQUIPMENT (In Last 3 Days) - Catheter |
| MANAGEMENT OF EQUIPMENT (In Last 3 Days) - Ostomy |
| VISITS IN LAST 90 DAYS OR SINCE LAST ASSESSMENT - Number of times ADMITTED TO HOSPITAL with an overnight stay |
| VISITS IN LAST 90 DAYS OR SINCE LAST ASSESSMENT - Number of times VISITED EMERGENCY ROOM without an overnight stay |
| VISITS IN LAST 90 DAYS OR SINCE LAST ASSESSMENT - EMERGENT CARE |
| TREATMENT GOALS - Any treatment goals that have been met in the LAST 90 DAYS (or since last assessment if less than 90 days) |
| OVERALL CHANGE IN CARE NEEDS - Overall self-sufficiency has changed significantly as compared to status of 90 DAYS AGO (or since last assessment if less than 90 days) |
| TRADE OFFS - Because of limited funds, during the last month, client made trade-offs among purchasing any of the following: prescribed medications, sufficient home heat, necessary physician care, adequate food, home care |
| **SECTION Q. MEDICATIONS** |
| NUMBER OF MEDICATIONS |
| RECEIPT OF PSYCHOTROPIC MEDICATION - Antipsychotic/neuroleptic |
| RECEIPT OF PSYCHOTROPIC MEDICATION - Anxiolytic |
| RECEIPT OF PSYCHOTROPIC MEDICATION - Antidepressant |
| RECEIPT OF PSYCHOTROPIC MEDICATION - Hypnotic |
| COMPLIANCE/ ADHERENCE WITH MEDICATIONS |
| **SCALES** |
| MAPLe (The Method of Assigning Priority Levels) |
| CHESS (The Changes in Health, End-Stage Disease, Signs, and Symptoms Scale) |
